# Autonomous surgical robotic systems and the liability dilemma

**DOI:** 10.3389/fsurg.2022.1015367

**Published:** 2022-09-16

**Authors:** Aimun A.B. Jamjoom, Ammer M.A. Jamjoom, Jeffrey P. Thomas, Paolo Palmisciano, Karen Kerr, Justin W. Collins, Effy Vayena, Danail Stoyanov, Hani J. Marcus, The iRobotSurgeon Collaboration

**Affiliations:** ^1^ Centre for Clinical Brain Sciences, Edinburgh University, Edinburgh, United Kingdom; ^2^ Department of Trauma and Orthopaedic Surgery, Leeds General Infirmary, Leeds, United Kingdom; ^3^ Department of Management, London School of Economics, London, United Kingdom; ^4^ Department of Neurosurgery, Trauma and Gamma Knife Center, Cannizzaro Hospital, Catania, Italy; ^5^ Digital Surgery, Medtronic Ltd, London, United Kingdom; ^6^ Division of Surgery and Interventional Science, Research Department of Targeted Intervention, University College London, London, United Kingdom; ^7^ Institute of Translational Medicine, ETH Zurich, Zurich, Switzerland; ^8^ Wellcome/EPSRC Centre for Interventional and Surgical Sciences (WEISS), University College London, London, United Kingdom; ^9^ Division of Neurosurgery, UCL Queen Square Institute of Neurology, University College London, London, United Kingdom

**Keywords:** robotics, surgery, liability, public opinion, autonomous

## Abstract

**Background:**

Advances in machine learning and robotics have allowed the development of increasingly autonomous robotic systems which are able to make decisions and learn from experience. This distribution of decision-making away from human supervision poses a legal challenge for determining liability.

**Methods:**

The iRobotSurgeon survey aimed to explore public opinion towards the issue of liability with robotic surgical systems. The survey included five hypothetical scenarios where a patient comes to harm and the respondent needs to determine who they believe is most responsible: the surgeon, the robot manufacturer, the hospital, or another party.

**Results:**

A total of 2,191 completed surveys were gathered evaluating 10,955 individual scenario responses from 78 countries spanning 6 continents. The survey demonstrated a pattern in which participants were sensitive to shifts from fully surgeon-controlled scenarios to scenarios in which robotic systems played a larger role in decision-making such that surgeons were blamed less. However, there was a limit to this shift with human surgeons still being ascribed blame in scenarios of autonomous robotic systems where humans had no role in decision-making. Importantly, there was no clear consensus among respondents where to allocate blame in the case of harm occurring from a fully autonomous system.

**Conclusions:**

The iRobotSurgeon Survey demonstrated a dilemma among respondents on who to blame when harm is caused by a fully autonomous surgical robotic system. Importantly, it also showed that the surgeon is ascribed blame even when they have had no role in decision-making which adds weight to concerns that human operators could act as “moral crumple zones” and bear the brunt of legal responsibility when a complex autonomous system causes harm.

## Introduction

Advances in machine learning and robotics have allowed the development of increasingly autonomous robotic systems which are able to make decisions and learn from experience. This distribution of decision-making away from human supervision poses a legal challenge for determining liability as these systems become more independent (1). This is particularly the case with surgical robotic systems due to the inherent risks posed by surgery. There are several human-controlled surgical robotic systems currently on the market, of which the da Vinci system (Intuitive Surgical, USA) is one of the best known. However, in recent years, more advanced systems have been developed that assist the surgeon, executing specific tasks such as navigating the gut, directing screw insertion into the spine or suturing (2–4). With the exponential pace of technological advancement, there is the prospect that a fully autonomous surgical robotic system will be developed in the not-so-distant future. The performance of such a system may prove superior to human surgeons bringing with it improvements in patient care and outcomes. However, with decision-making shifted away from the human surgeon and towards the robotic system, how do you ascribe liability if harm comes to the patient? Surveys of public opinion have found that the issue of legal liability with autonomous vehicles is a major worry (5). In particular, there is concern that there will be a bias towards human actors taking on a disproportionate burden of responsibility in complex human-robot systems; a situation which has been described as the human operator being the “moral crumple zone”, in the sense that humans may absorb the moral and legal responsibility when accidents occur, as the reputation of the technology is protected (6). We believe there is a need to explore public attitudes towards the issue of liability with surgical robotic systems (7). Here we report the findings of the iRobotSurgeon Survey focusing on how the burden of responsibility is allocated with increasing robotic system autonomy and the effect of demographic and geographic factors on these preferences.

## Materials and methods

### Development of the iRobotSurgeon survey

**Figure 1:**
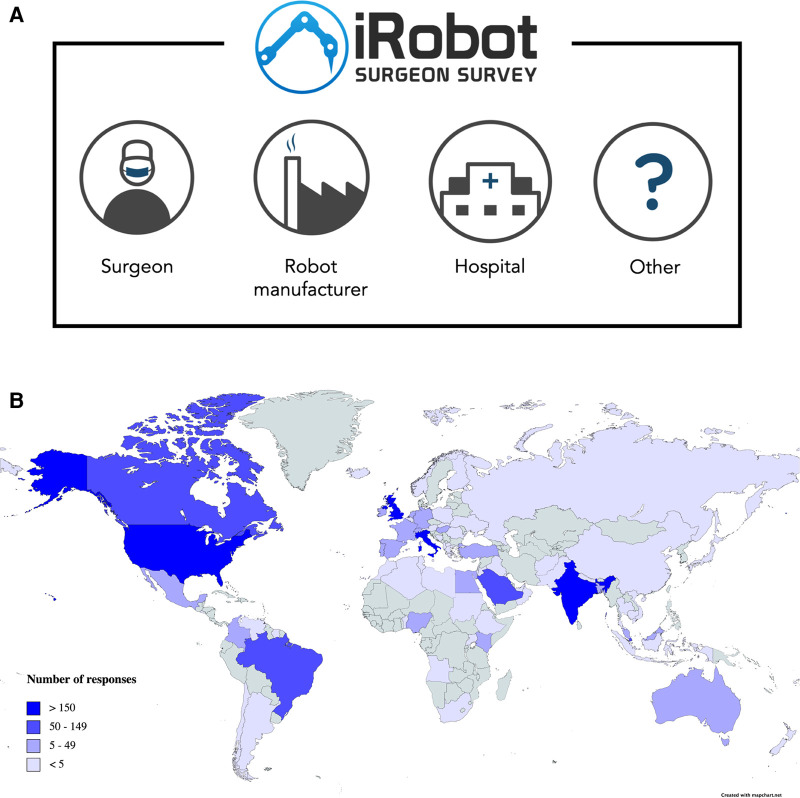
The irobotsurgeon survey poses five scenarios to respondents and asks them to decide who they believe is most liable: the surgeon, robot manufacturer, hospital or another party (**A**) a total of 2191 responses were collected from 78 countries spanning 6 continents (**B**).

The study was approved by the ethical board of the London School of Economic (Ref 08387). The iRobotSurgeon survey was developed through an iterative and consultative process with a range of stakeholders including clinicians, ethicists, members of industry and public engagement professionals. Individual scenarios were designed to test attitudes towards increasing robotic system autonomy with decision-making across three domains (management decision, operative planning and technical execution) shifting away from the human surgeon (Table 1). The definitions of these decisions were: management decision (the primary party deciding the need for surgery to treat the underlying pathology), operative planning (the primary party deciding on the type of surgery and how it should be approached) and technical execution (the primary party making intraoperative technical decisions such as where/what to cut). A test survey was posed to a group of patients and their relatives to get feedback on the survey's content, language, and ways to improve it (Supplementary material). Once finalised, the survey included five hypothetical scenarios where a patient comes to harm and the respondent needs to determine who they believe is most responsible: the surgeon, the robot manufacturer, the hospital, or another party (Figure 1A). Several demographic parameters were collected including age, gender, country, education level, occupation and if the respondent had previously undergone surgery. At the end of the survey a simple question was posed to the respondent as an attention check.

**Table 1:** Five scenarios describing robotic surgical systems with increasing autonomy.

			Primary decision maker		
Level of autonomy	Scenario		Management decision	Operative planning	Technical execution
**Level 1 - Human-controlled robotic system:** these systems include robots that are completely controlled by the surgeon who can sometimes operate remotely (telesurgical robot). Other robots are integrated within handheld instruments and may, for example, warn the doctor when they are operating close to important parts of the body (handheld robot).	**Scenario 1**	A world-leading heart surgeon (Surgeon A) operates remotely on a patient in a different country using a telesurgical system. During the operation, a major blood vessel is cut open. Surgeon A cannot stop the bleeding using the robot. A support surgeon in the operating room (Surgeon B) steps in and controls the bleeding. Despite this, the patient loses blood and is harmed.	Surgeon	Surgeon	Surgeon
** **	**Scenario 2**	A surgeon uses a robotic telescope while operating on a patient. Its purpose is to inform the surgeon about the location of an important blood vessel. The surgeon plans to use this information and their knowledge of anatomy to perform the operation safely. During surgery, the robot malfunctions. It gives the surgeon inaccurate information. The blood vessel is cut and the patient is harmed.	Surgeon	Surgeon	Surgeon
**Level 2 - Robot-assisted system**: these systems help the surgeon carry out specific tasks. This could be stitching wounds, inserting a needle into the brain, or inserting a screw to fix a broken bone. The surgeon is present and supervises the robot.	**Scenario 3**	A patient has an operation where screws are inserted into the bone of their spine by a robot. A surgeon pre-programmes the robot with directions for the screws to be fixed. The robot then carries out the operation independently as the surgeon supervises. After the operation, the patient wakes up and cannot move their legs. A follow-up scan shows a screw has been put into the wrong place, causing spinal injury. An investigation finds the surgeon had correctly programmed the robot, directing the screws away from the spinal cord.	Surgeon	Surgeon	Robotic system
**Level 3 - Autonomous robotic system**: this system can conduct entire surgical procedures with minimal or no human supervision.	**Scenario 4**	A surgeon recommends a hip replacement operation for a patient. A robot carries out the surgery independently and the surgeon, who supervises, does not intervene. The operation is technically successful and follow-up scans show that the hip was repaired as planned. However, the patient is left with worse hip pain which badly affects their quality of life.	Surgeon	Robotic system	Robotic system
	**Scenario 5**	An intelligent robot develops a new surgical technique to treat pancreatic cancer. Research through clinical trials shows the new technique is better than existing treatments. A surgeon refers a patient with newly diagnosed pancreatic cancer for the procedure. During the operation, the robot cannot manage a complication in the surgery and the patient is harmed.	Robotic system	Robotic system	Robotic system

The three levels of autonomy described by Jamjoom et al. (7). The table also demonstrates a comparison of the primary decision makers between the surgeon and the robotic system. Patient management is broken down into three stages: management decision (the primary party deciding the need for surgery to treat the underlying pathology), operative planning (the primary party deciding on the type of surgery and how it should be approached) and technical execution (the primary party making intraoperative technical decisions such as where/what to cut).

### Distribution of the iRobotSurgeon survey

The survey was launched on 1 January 2020 and was open for 1 year till 31 December 2020. The survey was delivered using the SurveyMonkey™ platform. The survey was distributed through two approaches. The first was through social media and messaging networks (Twitter Inc, Facebook Inc and Watsapp by Facebook Inc) through a collaborator-led model. Study collaborators were recruited to assist with the study by distributing the survey through their social networks. The second approach was through a compensation-per-response approach performed through the Amazon Mechanical Turk (mTurk) platform. Respondents were paid $1 per response.

### Data analysis

Responses were included in the final analysis if all questions had been answered and had a correct answer to the attention check question at the end of the survey. Concordance analysis be internal validity was performed using mTurk responses where individual respondents could be identified using their mTurk numbers. Those respondents who responded more than once were used to check if there was concordance between their individual scenario responses. Concordance was defined as agreement between each of their responses for any given scenario. Two-tailed Pearson correlations between variables was performed. Linear Ordinary Least Squares (OLS) regression models were developed to test the explanatory power of multiple demographic variables including having had surgery in the past, gender, age range, education level, and profession. Statistical analysis and graphical representation were performed using GraphPad Prism 9.1.0 (216. GraphPad Software, LLC.) and SPSS 24 (IBM Corp. Released 2017. IBM SPSS Statistics for Windows, Version 25.0. Armonk, NY: IBM Corp).

## Results

**Figure 2:**
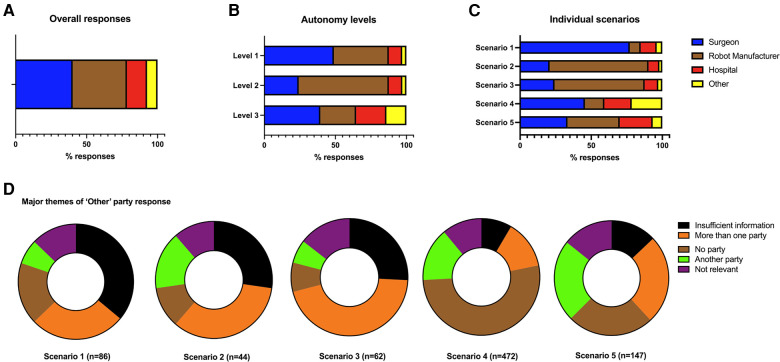
A total of 10,955 individual scenario responses were captured from 2,191 respondents. Bar charts demonstrating the overall responses across the five scenarios (**A**) the levels of autonomy (**B**) and the individual scenarios (**C**). The comments from another party option were collated and qualitatively then quantitively analysed (**D**).

A total of 2,191 completed surveys were gathered evaluating 10,955 individual scenario responses from 78 countries spanning 6 continents (Figure 1B). Basic demographic data was collected from survey respondents including the country they were living in (Supplementary materials). From the 10,955 responses, surgeons (*n* = 4,404, 40.2%) were the most identified responsible party across the five scenarios. This was followed by robot manufacturer (*n* = 4,186, 38.2%), the hospital (*n* = 1,554, 14.2%) and another party (*n* = 811; 7.4%) (Figure 2A). When respondents allocated blame to “other” they were invited to leave a text comment. These comments were qualitatively analysed, and five themes emerged: no party is responsible (*n* = 308; 37.9%), one or more party is responsible (*n* = 166; 20.5%), another party is responsible (*n* = 121; 14.9%), more information required to decide (*n* = 118; 14.5%) and not relevant comments (*n* = 98; 12.1%) (Figure 2D).

### Blame distribution across levels of autonomy and scenarios

The individual scenarios were then categorised based upon the degree of the surgical robotic system's autonomy level based upon a three-level classification described by Jamjoom et al. (Table 1) (7). For level 1 robotic systems, where the human surgeon is the primary decision maker across all parts of the patient management process, the surgeon was the most commonly ascribed responsible party with 2,145 (48.9%) of respondents placing the blame with them (Figure 2B). For level 2 systems where the robotic system is starting to take on more autonomy by technically executing an operative plan defined by the surgeon, the robot manufacturer was the most commonly identified responsible party (*n* = 1,390; 63.4%) in the situation a technical malfunction leads to patient harm. For level 3 systems where most or all of the decision-making was taken on by the surgical robotic system, there was no clear consensus on who shoulder responsibility: with surgeon responsibility at (*n* = 1,729, 39.5%), robot manufacturer responsibility at (*n* = 1,103, 25.1%) and hospital responsibility at (*n* = 931, 21.3%).

Looking at individual scenarios demonstrated interesting insights into how the distribution of decision-making impacts perceptions on who holds responsibility (Figure 2C). In scenario 1, a patient comes to harm after a complication arises during an operation performed by a surgeon (Surgeon A) using a tele-robotic system despite the efforts of a support surgeon (Surgeon B). This system is completely under control of the surgeon who is the primary decision maker on management approach and technical execution. In this scenario, a clear majority (*n* = 1,482; 67.6%) identified Surgeon A as the primary bearer of responsibility. A one-sample chi-square analysis showed a statistically significant deviation from equal distribution across 5 blame categories in Scenario 1, (*χ*^2^(4) = 3,140.03, *p* < .01). By contrast, in scenario 2, where a patient comes to harm due to a smart robotic telescope providing inaccurate information to the surgeon, the majority of respondents (*n* = 1,524; 69.6%) view was that the robot manufacturer was most at blame despite the surgeon being the primary decision maker for the patient's management (*χ*^2^(3) = 2,482.22, *p* < .01). Similarly in scenario 3, a patient comes to harm due to technical malfunction of the robotic system despite a correctly planned procedure by the surgeon. In this circumstance, a majority (*n* = 1,390; 63.4%) felt that the robot manufacturer was most responsible (*χ*^2^(3) = 1,935.92, *p* < .01). In scenario 4, a patient does not get a satisfactory outcome after an operative performed independently by a surgical robotic system based upon the decision for surgery by a human surgeon. In this case, no party reached over 50% consensus of respondents, but the surgeon was attributed responsibility by 998 (45.6%) respondents (*χ*^2^(3) = 521.96, *p* < .01). Finally, in scenario 5, the surgical robotic system makes all the decisions on how the patient is managed and executes the operation. In this scenario, there was a relatively equal distribution of blame ascribed across all three parties: robot manufacturer (*n* = 803; 36.7%), surgeon (*n* = 731; 33.4%) and the hospital (*n* = 510; 23.3%) (*χ*^2^(3) = 476.05, *p* < .01).

### Effect of demographics on blame distribution

In terms of effect of demographics on blame allocation, experience with past surgery was negatively correlated with robot blame (*r* = −.06, *p* < .01), and positively correlated with hospital blame (*r* = .05, *p* < .01). Female respondents were more likely to select the “other” category as the target of blame (*r* = .08, *p* < .01) (Table 2). Employment in the healthcare domain was negatively correlated with robot blame (*r* = −.12; *p* < .05), and positively correlated with surgeon blame (*r* = .09, *p* < .01). Age was negatively correlated with robot blame and positively correlated with “other” blame. Education level was negatively correlated with robot blame (*r* = −.12, *p* < .05) and positively correlated with surgeon blame (*r* = .08, *p* < .01). Utilizing linear Ordinary Least Squares (OLS) regression analysis, two models were developed predicting total blame distribution across the 5 scenarios (Table 3). Analyses revealed significant predictors of robot blame in Model 1, with past surgery (*b* = −.05, *t* = −2.76, *p* < .01), age (*b* = −.04, *t* = −2.21, *p* < .01), education level (*b* = −.07, *t* = −2.82, *p* < .01), and employment in healthcare (*b* = −.09, *t* = −3.49, *p* < .01) being negatively related to robot blame. In Model 2 predicting surgeon blame, we found that women were less likely to blame surgeons (*b* = −.04, *t* = −2.01, *p* < .05) while there was an increase in surgeon blame associated with occupation in domains including business (*b* = .08, *t* = 3.39, *p* < .01), computing (*b* = .05, *t* = 2.17, *p* < .05), and healthcare (*b* = .11, *t* = 4.17, *p* < .01).

**Table 2:** Two-tailed Pearson correlations among study variables (*n* = 2,191).

	1	2	3	4	5	6	7	8	9	10	11
1. Total robot blame	–										
2. Total surgeon blame	−.52**	–									
3. Total hospital blame	−.39*	−.37**	–								
4. Total other blame	−.12**	−.34**	−.16**	–							
5. Age range	−.06**	.03	−.03	.09**	–						
6. Gender	.01	−.04	.00	.08**	.03	–					
7. Past Surgery	−.06**	.01	.05**	.00	.11**	.02	–				
8. Profession business	.04*	.02	−.02	−.08**	.11**	−.02	.03	–			
9. Profession computing	−.01	.00	.03	−.03	.00	−.10**	.00	−.24**	–		
10. Profession healthcare	−.12**	.09**	−.03	.09**	.02	.06**	.00	−.34**	−.27**	–	
11. Profession other	.08**	−.11**	.03	.01	−.12**	.03	−.01	−.37**	−.30**	−.42**	–
12. Education level	−.12**	.08**	−.02	.07**	.08**	.00	−.04	−.15**	−.47*	.50**	−.30**

*,*p *< .05, **p < .01.

**Table 3:** Linear regression predicting blame across scenarios.

	Robot blame	Surgeon blame	Hospital blame	Other/No blame
Past Surgery	−.05 (−2.76)**	.01 (0.55)	.06 (2.87)**	−.01 (0.71)
Gender (0 = Male; 1 = Female)	−.01 (−0.56)	−.04 (−2.01)*	.01 (.056)	.07 (3.49)**
Age range	−.04 (−2.21)**	.01 (0.87)	−.03 (−1.75)	.09 (4.52)**
Education Level	−.07 (−2.82)**	.04 (1.63)	−.00 (−.07)	.04 (1.77)
Profession Business	−.00 (0.01)	.08 (3.39)**	−.03 (−1.38)	−.09 (−3.77)**
Profession Computing	−.04 (−1.65)	.05 (2.17)*	.01 (0.50)	−.04 (−1.82)
Profession Healthcare	−.09 (−3.49)**	.11 (4.17)**	−.04 (−1.57)	.01 (0.66)
Overall R	*R* = .16 *F*(7,2183) = 8.89**	*R* = .13 *F*(7,2183)= 6.15**	*R* = .08 *F*(7,2183) = 2.50*	*R* = .17 *F*(7,2183) = 9.67**

Regression predicting total blame distribution across 5 scenarios. Standardized regression beta values presented, *t* values presented in parentheses.

*p < .05, **p < .01.

## Discussion

Our findings suggest a dilemma on how to ascribe responsibility with increasing autonomy with surgical robotic systems. As more decision-making is taken on by the robotic system across the patient management process, there is a growing divergence of opinion on who shoulders the responsibility when the system fails. When a patient came to harm with the surgeon controlling the robotic system then the surgeon was viewed as the most responsible. This supports the finding by Furlough and colleagues who found that human actors received most blame in scenarios of non-autonomous robotic systems (8). Conversely, in the event of a technical fault with the robotic system, be it providing inaccurate information to the surgeon or not executing a pre-planned operation correctly, there was consensus from the respondents that the robot manufacturer is most responsible in these situations. However, with autonomous systems, there were no clear majorities on where to allocate the blame. This reflects a growing uncertainty and disagreement among the respondents on who to ascribe responsibility to. Saying that, the surgeon is still the most identified responsible actor despite them having a limited role in the decision-making process for the patients' care across the two scenarios. This adds weight to the concept of the “moral crumple zone” which was coined by Madeline Elish to describe humans bearing the consequences of failure in complex human-robot systems (6). This has been shown quantitively by Awad and colleagues who examined blame distribution in semi-autonomous vehicle accidents and found that where a human and machine share control of a car, more blame is ascribed to the human driver when both drivers make an error (9). These findings represent evidence of bias in how the public distribute blame in autonomous systems, however there is also legal precedent in medicine that tilts liability away from the manufacturer and towards the physician. The “Learned Intermediary” doctrine limits the recovery from a manufacturer when they have provided adequate information about the risks of their device or drug (10).

Machine learning approaches permit robotic systems to learn and solve problems with solutions previously unknown to human operators. This degree of independence in decision-making, as with scenario 5, can lead to unpredictable actions which poses significant legal challenges in determining liability. Firstly, in Tort law, negligence is an action that leads to unreasonable harm or risk to property or an individual (11). As these systems becomes more autonomous and move away from predetermined instructions and arrive at novel decisions, it becomes difficult to determine and define this standard. This “black box” challenge in machine learning has been the driving force behind calls for making the solutions reached by these algorithms explainable to humans. Hacker and colleagues argue that current tort liability provides incentives to make machine learning algorithms explainable to protect professional actors such as doctors (12). This view is backed up by a study by Kim and Hinds who found a reduction in blame attribution to human participants when autonomous robotic system was more transparent in their decision-making (13). Our finding that the public has a bias towards ascribing blame to the surgeon, even when they have limited role in decision-making, has important policy implications. As suggested by Awad et al (9), this finding highlights that a bottom-up regulatory system from Tort law adjudicated in a jury system may fail to regulate complex autonomous systems effectively. A recently published WHO guidance on the governance of artificial intelligence for health recommended establishing international norms and legal standards to ensure national accountability to protect patients (14).

In this study, a number of demographic factors appeared to influence respondents' likelihood to attribute blame to the surgeon. This included male respondents and those working in healthcare, computing and business professions. Conversely, robot manufacturer blame was reduced in those respondents who had experienced surgery, were older, had higher levels of education and worked in healthcare. This highlights that those with experience of the healthcare system, be it as a patient or professionally, tend to direct blame towards surgeon as opposed to the robot manufacturer. For the healthcare professionals, this is may be explained by the respondents' understanding that the onus of responsibility typically lies with the surgeon. While for respondents who had previously undergone surgery, the experience of placing trust in a surgeon may have influenced their perception of blame attribution in the study.

The study has several limitations which need to be considered when drawing conclusions from our results. Firstly, the respondent population was titled towards a more male, medically trained and highly educated population. In conjunction to this, despite responses from a wide range of countries, there were 27 countries with only 1 response which limits the generalisability of the findings. The scenarios posed to respondents had limited information in them which several respondents felt was insufficient to make a decision as reflected in some of the text responses. Coupled to this, our decision to use categorical answers prevented respondents from ascribing proportions of blame to multiple parties.

## Conclusion

As decision-making becomes distributed across increasingly intelligent autonomous systems there is a growing challenge in determining liability. In this study, we provide the first empirical evidence of current public attitudes towards this problem and demonstrate a liability dilemma as surgical robotic systems become increasingly autonomous. This highlights the challenges facing policy makers and regulators in developing legal frameworks around these new technologies, but also the importance of engaging the public with this process.

## Data Availability

The iRobotSurgeon raw data is available from the Edinburgh University's Data Share service via: https://datashare.ed.ac.uk/handle/10283/4481.

## iRobotSurgeon Collaborators

D. Dasgupta, Y.J. Tan, Y.T. Lo, A. Asif, F. Samad, A. Negida, R. McNicholas, L. McColm, A. Chari, H. Al Abdulsalam, S. Venturini, T. Hasan, G.H. Majernik, S. Sravanam, B. Vasey, D. Taylor, H. Lambert.
