# Supplementary material for: Autonomous surgical robotic systems and the liability dilemma
Source: Front Surg. 2022 Sep 16;9:1015367. doi: 10.3389/fsurg.2022.1015367 (PMC9580336; doi:10.3389/fsurg.2022.1015367)
Supplement: Supplementary file 1 [file Datasheet1.pdf]

SUPPLEMENTARY MATERIAL

Table of content	Page
Public consultation	2
Response rate	4
Internal validity	4
Demographic data	5
Comparison of demographic characteristic between mTurk and social media respondents	9
Responses for individual scenarios	11
Overall responses (across all 5 scenarios)	13
Effect of demographic parameters on overall responses	14
Autonomy level analysis	16
Survey sample	18

Public consultation

A cross-sectional mixed qualitative and quantitative survey was performed to appraise the publics' understanding of and attitudes towards the scenarios proposed to be presented in the iRobot Surgeon Survey. The survey was devised on SurveyMonkey (Momentive Global Inc., San Mateo, CA, USA) observing the guidelines of good practice in conduct and reporting of survey research. The survey was distributed over a two-week period of October 2019 at the Department of Neurosurgery of the senior author's (H.J.M.) institution and administered in person using a tablet computer (iPad; Apple Inc., Cupertino, California, USA). A total of 11 participants were recruited from inpatients and their relatives, using the following inclusion criteria: 1) adequate capacity to understand and complete the survey 2) willingness to participate. The public consultation survey was organized into three sections for each one of the 5 proposed scenarios. First, the clinical scenario was described, and the available response options were presented. Second, participants were asked to rate how much the scenario was *understandable* (how much they could understand by reading its description), *concerning* (how much they would be worried if that scenario would present in real-life settings), and *answerable* (if the available response options were sufficient to answer the question). Responses were rated using 5-point Likert-scales, from 0 = "not at all" to 4 = "very much" (Table 1). Third, two open-ended questions were asked: 1) "Do you have any concerns about this scenario being presented to the general public? If yes, please state why."; 2) "Do you have any recommendations on ways to improve the scenario?". The free text responses were analysed qualitatively and themes were identified and categorised (Table 2).

Table 1: Results from public consultation for each individual scenario

Scenarios	Understandable		Concerning		Answerable	
	Option	%	Option	%	Option	%
1	0(not at all)	0%	0(not at all)	0%	0(not at all)	45.4%
	1	0%	1	0%	1	36.4%
	2	0%	2	18.2%	2	0%
	3	27.3%	3	9.1%	3	27.3%
	4(very much)	72.3%	4(very much)	72.3%	4(very much)	9.1%
2	0(not at all)	0%	0(not at all)	0%	0(not at all)	18.2%
	1	0%	1	0%	1	18.2%
	2	0%	2	0%	2	18.2%
	3	36.4%	3	36.4%	3	27.3%
	4(very much)	63.3%	4(very much)	63.6%	4(very much)	18.2%
3	0(not at all)	0%	0(not at all)	0%	0(not at all)	18.2%
	1	9.1%	1	0%	1	0%
	2	18.2%	2	0%	2	9.1%
	3	18.2%	3	9.1%	3	27.3%

	4(very much)	54.5%	4(very much)	90.9%	4(very much)	36.4%
4	0(not at all)	0%	0(not at all)	0%	0(not at all)	9.1%
	1	0%	1	0%	1	18.2%
	2	0%	2	36.4%	2	9.1%
	3	36.4%	3	27.3%	3	18.2%
	4(very much)	63.3%	4(very much)	36.4%	4(very much)	45.4%
5	0(not at all)	0%	0(not at all)	0%	0(not at all)	18.2%
	1	0%	1	0%	1	9.1%
	2	0%	2	9.1%	2	36.4%
	3	36.4%	3	9.1%	3	9.1%
	4(very much)	63.3%	4(very much)	81.8%	4(very much)	27.3%

Table 2: Summary of key themes from free text responses

**Concerns about presenting these scenarios to the public**	**No**	**%**
(1) The human element during surgery is not specified in detail	6/11	54.55%
(2) The information provided is not sufficient	5/11	45.45%
(3) Some mistakes may happen even without robots	5/11	45.45%
(4) The public mistrust towards robots may influence responses	4/11	36.36%
(5) Bad hospital maintenance may be confused with manufacturer errors	2/11	18.18%
**Recommendations for improving the public understanding**	**No**	**%**
(1) Provide more information on the role of the surgeon	8/11	72.73%
(2) Add the extra response option “jointed responsibility of the surgeon and the robot”	7/11	63.64%
(3) Provide more general information on robotics and medicine	2/11	18.18%

Response rate

Duplicates from mTurk were removed. Completed questionnaires were defined as questionnaires with completed attention question at end of questionnaire. Responses were included in final data set if respondent got attention question correct at end of survey

Cohort	No	%
**All responses**	**2673**	**100.0**
Duplicates removed	2554	95.6
Completed questionnaires	2293	85.8
Included data	2191	82.0

Internal validity

A total of 90 mTurk respondents completed a questionnaire more than once. This data was used to determine concordance between responses from individual respondents. Concordance was defined as complete agreement across all responses for specific scenario by an individual respondent.

	Concordant (n)	%
**Total**	**301**	**66.89%**
Scenario 1	66	73.33%
Scenario 2	68	75.56%
Scenario 3	64	71.11%
Scenario 4	45	50.00%
Scenario 5	58	64.44%

Demographic data

Age

Age Group	No of responses	
	No.	%
**Grand Total**	**2191**	**100.00%**
25-34	1024	46.55%
35-44	440	20.08%
18-24	363	16.56%
45-54	184	8.40%
55-64	135	6.16%
65+	49	2.24%

Gender

Gender	No of responses	
	No.	%
**Grand Total**	**2191**	**100.00%**
Male	1367	62.39%
Female	824	37.61%

Country

Country	No of responses	
	No.	%
**Grand Total**	**2191**	**100.00%**
United States of America	540	24.65%
United Kingdom of Great Britain and Northern Ireland	372	16.98%
India	341	15.56%
Italy	154	7.03%
Brazil	139	6.34%
Canada	131	5.98%
Singapore	73	3.33%
Saudi Arabia	58	2.65%
Malaysia	41	1.87%
Spain	39	1.78%
Germany	32	1.46%

Egypt	31	1.41%
France	19	0.87%
Australia	17	0.78%
Kenya	17	0.78%
Turkey	16	0.73%
Netherlands	14	0.64%
Hungary	9	0.41%
Ireland	9	0.41%
Mexico	9	0.41%
Switzerland	9	0.41%
Bangladesh	6	0.27%
Nigeria	6	0.27%
Portugal	6	0.27%
Colombia	5	0.23%
Argentina	4	0.18%
Austria	4	0.18%
China	4	0.18%
Ethiopia	4	0.18%
Romania	4	0.18%
Venezuela (Bolivarian Republic of)	4	0.18%
Afghanistan	3	0.14%
Algeria	3	0.14%
Belgium	3	0.14%
Greece	3	0.14%
Jordan	3	0.14%
New Zealand	3	0.14%
Poland	3	0.14%
Andorra	2	0.09%
Chile	2	0.09%
Finland	2	0.09%
Indonesia	2	0.09%
Israel	2	0.09%
Kuwait	2	0.09%
Libya	2	0.09%
Norway	2	0.09%
Pakistan	2	0.09%
South Africa	2	0.09%
Trinidad and Tobago	2	0.09%
Ukraine	2	0.09%
United Arab Emirates	2	0.09%
Albania	1	0.05%
Angola	1	0.05%

Anguilla	1	0.05%
Bahamas	1	0.05%
Barbados	1	0.05%
Benin	1	0.05%
Bosnia and Herzegovina	1	0.05%
Cambodia	1	0.05%
El Salvador	1	0.05%
Fiji	1	0.05%
Iceland	1	0.05%
Iraq	1	0.05%
Japan	1	0.05%
Luxembourg	1	0.05%
Malta	1	0.05%
Morocco	1	0.05%
Nepal	1	0.05%
Oman	1	0.05%
Russian Federation	1	0.05%
Slovakia	1	0.05%
Sudan	1	0.05%
Suriname	1	0.05%
Thailand	1	0.05%
The former Yugoslav Republic of Macedonia	1	0.05%
Tunisia	1	0.05%
Uganda	1	0.05%
Vietnam	1	0.05%

Occupation

Row Labels	Count of Start Date	%
Other	695	31.72%
Healthcare	618	28.21%
Business, finance and management	518	23.64%
Computing, science and engineering	360	16.43%

Education

Highest Educational Qualification	No of responses	
	No.	%
**Grand Total**	**2191**	**100.00%**
Bachelor's Degree	904	41.26%
Master's Degree	508	23.19%
A-level/IB/Equivalent high-school diploma	369	16.84%
Medical Degree	284	12.96%
PhD	126	5.75%

Previous Surgery

Previous Surgery	No of responses	
	No.	%
**Grand Total**	**2191**	**100.00%**
Yes	1280	58.42%
No	911	41.58%

Social vs mTurk

Source of response	No of responses	
	No	%
**Grand Total**	**2191**	**100.00%**
mTurk	1232	56.23%
Social	959	43.77%

Comparison of demographic characteristic between mTurk and social media respondents

Analysis was performed to examine whether there were significant differences the demographic characteristics of the mTurk (n=1232) and social media (n=959) cohorts. Chi-squared test was used to assess for significance.

Age

Row Labels	mTurk		Social		Total	
	No	%	No	%	No	%
**Grand total**	**1232**	**56.23%**	**959**	**43.77%**	**2191**	**100.00%**
18-24	172	7.85%	191	8.72%	363	16.57%
25-34	605	27.61%	415	18.94%	1020	46.55%
35-44	277	12.64%	163	7.44%	440	20.08%
45-54	102	4.66%	82	3.74%	184	8.40%
55-64	57	2.60%	78	3.56%	135	6.16%
65+	19	0.87%	30	1.37%	49	2.24%
**P value**	**<0.0001**					

Gender

Row Labels	mTurk		Social		Total	
	No	%	No	%	No	%
**Grand total**	**1232**	**56.23%**	**959**	**43.77%**	**2191**	**100.00%**
Female	416	18.99%	408	18.62%	824	62.39%
Male	816	37.24%	551	25.15%	1367	37.61%
**P value**	**<0.0001**					

Occupation

Row Labels	mTurk		Social		Total	
	No	%	No	%	No	%
**Grand total**	**1232**	**56.23%**	**959**	**43.77%**	**2191**	**100.00%**
Business, finance and management	395	18.03%	123	5.61%	518	23.64%
Computing, science and engineering	264	12.05%	96	4.38%	360	16.43%
Healthcare	193	8.81%	425	19.40%	618	28.21%
Other	380	17.34%	315	14.38%	695	31.72%
**P value**	**<0.0001**					

Education

Row Labels	mTurk		Social		Total	
	No	%	No		No	%
**Grand total**	**1232**	**56.23%**	**959**	**43.77%**	**2191**	**100.00%**
A-level/IB/Equivalent high-school diploma	211	9.63%	158	7.21%	369	16.84%
Bachelor’s Degree	666	30.40%	238	10.86%	904	41.26%
Master’s Degree	292	13.33%	216	9.86%	508	23.19%
Medical Degree	41	1.87%	243	11.09%	284	12.96%
PhD	22	1.00%	104	4.75%	126	5.75%
**P value**	**<0.0001**					

Previous Surgery

Row Labels	mTurk		Social		Total	
	No	%	No		No	%
**Grand total**	**1232**	**56.23%**	**959**	**43.77%**	**2191**	**100.00%**
No	423	19.31%	488	22.27%	911	41.58%
Yes	809	36.92%	471	21.50%	1280	58.42%
**P value**	**<0.0001**					

Response for individual scenarios

Responses to each scenario from the total cohort (n=2191)

Scenario 1

A world-leading heart surgeon (Surgeon A) operates remotely on a patient in a different country using a telesurgical system. During the operation, a major blood vessel is cut open. Surgeon A cannot stop the bleeding using the robot. A support surgeon in the operating room (Surgeon B) steps in and controls the bleeding. Despite this, the patient loses blood and is harmed.

Choices	No of responses	
	No.	%
**Grand Total**	**2191**	**100.00%**
Surgeon A	1482	67.64%
Hospital	246	11.23%
Surgeon B	208	9.49%
Robot manufacturer	169	7.71%
Other	86	3.93%

Scenario 2

A surgeon uses a robotic telescope while operating on a patient. Its purpose is to inform the surgeon about the location of an important blood vessel. The surgeon plans to use this information and their knowledge of anatomy to perform the operation safely. During surgery, the robot malfunctions. It gives the surgeon inaccurate information. The blood vessel is cut and the patient is harmed.

Choices	No of responses	
	No.	%
**Grand Total**	**2191**	**100.00%**
Robot manufacturer	1524	69.56%
Surgeon	455	20.77%
Hospital	168	7.67%
Other	44	2.01%

Scenario 3

A patient has an operation where screws are inserted into the bone of their spine by a robot. A surgeon pre-programmes the robot with directions for the screws to be fixed. The robot then carries out the operation independently as the surgeon supervises. After the operation, the patient wakes up and cannot move their legs. A follow-up scan shows a screw has been put into the wrong place, causing spinal injury. An investigation finds the surgeon had correctly programmed the robot, directing the screws away from the spinal cord.

Choices	No of responses	
	No.	%
**Grand Total**	**2191**	**100.00%**
Robot manufacturer	1390	63.44%
Surgeon	530	24.19%
Hospital	209	9.54%
Other	62	2.82%

Scenario 4

A surgeon recommends a hip replacement operation for a patient. A robot carries out the surgery independently and the surgeon, who supervises, does not intervene. The operation is technically successful and follow-up scans show that the hip was repaired as planned. However, the patient is left with worse hip pain which badly affects their quality of life.

Choices	No of responses	
	No.	%
**Grand Total**	**2191**	**100.00%**
Surgeon	998	45.55%
Other	472	21.54%
Hospital	421	19.21%
Robot manufacturer	300	13.69%

Scenario 5

An intelligent robot develops a new surgical technique to treat pancreatic cancer. Research through clinical trials shows the new technique is better than existing treatments. A surgeon refers a patient with newly diagnosed pancreatic cancer for the procedure. During the operation, the robot cannot manage a complication in the surgery and the patient is harmed.

Choices	No of responses	
	No.	%
**Grand Total**	**2191**	**100.00%**
Robot manufacturer	803	36.65%
Surgeon	731	33.36%
Hospital	510	23.28%
Other	147	6.71%

Overall responses (across all 5 scenarios)

All Data

Aggregate responses across all 5 scenarios from n=2191 respondents providing a total of 10955 individual scenario responses

Option	Q1	Q2	Q3	Q4	Q5	Total	%
**Total**	**2191**	**2191**	**2191**	**2191**	**2191**	**10955**	**100.00%**
Surgeon (A+B for Q1)	1690	455	530	998	731	4404	40.20%
Robot Manufacturer	169	1524	1390	300	803	4186	38.21%
Hospital	246	168	209	421	510	1554	14.19%
Other	86	44	62	472	147	811	7.40%

Effect of demographic parameters on overall responses

Total responses across the 5 scenarios analysed based upon 7 demographic parameters. Statistical significance tested with Chi-squared test.

Age

Age group	Hospital	Other	Robot manufacturer	Surgeon
18-24	268	129	758	660
25-34	748	319	1927	2106
35-44	299	169	844	888
45-54	129	95	318	378
55-64	81	74	257	263
65+	29	25	82	109
**P value**	**<0.0001**			

Gender

Row Labels	Hospital	Other	Robot manufacturer	Surgeon
Female	594	368	1554	1604
Male	960	443	2632	2800
**P value**	**<0.0001**			

Occupation

Row Labels	Hospital	Other	Robot manufacturer	Surgeon
Business, finance and management	345	135	1039	1071
Computing, science and engineering	281	113	681	725
Healthcare	401	293	1046	1350
Other	527	270	1420	1258
**P value**	**<0.0001**			

Education

Row Labels	Hospital	Other	Robot manufacturer	Surgeon
A-level/IB/Equivalent high-school diploma	240	145	787	673
Bachelor’s Degree	699	270	1771	1780
Master’s Degree	364	174	918	1084

Medical Degree	159	158	494	609
PhD	92	64	216	258
**P value**	**<0.0001**			

Previous Surgery

Row Labels	Hospital	Other	Robot manufacturer	Surgeon
No	585	342	1813	1815
Yes	969	469	2373	2589
**P value**	**0.0015**			

Social vs mTurk

Row Labels	Hospital	Other	Robot manufacturer	Surgeon
mTurk	863	276	2516	2505
Social	691	535	1670	1899
**P value**	**<0.0001**			

Autonomy level analysis

Scenarios were classified based upon the level of robotic autonomy

Level 1

Human-controlled robotic system: these systems include robots that are completely controlled by the surgeon who can sometimes be in a different place to the surgery (telesurgical robot). Other robots are integrated within handheld instruments and may, for example, warn the doctor when they are operating close to important parts of the body (handheld robot).

Option	Q1	Q2	Total	Percentage
**Total**	**2191**	**2191**	**4382**	**100.00%**
Surgeon	1690	455	2145	48.95%
Robot Manufacturer	169	1524	1693	38.64%
Hospital	246	168	414	9.45%
Other	86	44	130	2.97%

Level 2

Robot-assisted system: these systems help the surgeon carry out specific tasks. This could be stitching wounds, inserting a needle into the brain, or inserting a screw to fix a broken bone. The surgeon is present and supervises the robot.

Option	Q3	Percentage
**Total**	**2191**	**100.00%**
Robot Manufacturer	1390	63.44%
Surgeon	530	24.19%
Hospital	209	9.54%
Other	62	2.83%

Level 3

Autonomous robotic system: this system can conduct entire surgical procedures with minimal or no human supervision.

Option	Q4	Q5	Total	Percentage
**Total**	**2191**	**2191**	**4382**	**100.00%**
Surgeon	998	731	1729	39.46%
Robot Manufacturer	300	803	1103	25.17%
Hospital	421	510	931	21.25%
Other	472	147	619	14.13%

Survey sample

Demographic information

Which of these age groups do you belong to?
15-30
30-45
45-60
60-75
>75

What is your gender?
Male
Female

In what country do you live?
<full list of nations>

Which choice best describes your occupation?
Medical - Surgeon
Medical – Non-surgical
Other healthcare
Life, Physical, and Social Science Occupations
Sales and Related Occupations
Business and Financial Operations Occupations
Personal Care and Service Occupations
Computer and Mathematical Occupations
Construction and Extraction Occupations
Legal Occupations
Education, Training, and Library Occupations
Farming, Fishing, and Forestry Occupations
Food Preparation and Serving Related Occupations
Protective Service Occupations
Office and Administrative Support Occupations
Building and Grounds Cleaning and Maintenance Occupations
Arts, Design, Entertainment, Sports, and Media Occupations
Installation, Maintenance, and Repair Occupations
Architecture and Engineering Occupations
Community and Social Service Occupations
Management Occupations
Production Occupations
Student
Transportation and Materials Moving Occupations
Not stated above

What is your highest academic qualification?
A-level/IB/Equivalent high-school diploma
Bachelor's Degree
Master's Degree
PhD

Have you had surgery in the past?
Yes
No

MTurk respondents only: Please provide your Worker ID number?

Case 1

Level 1: Human-controlled robot system

A world-leading heart surgeon (surgeon A) operates remotely on a patient in a different country using a telesurgical system. During the operation, a major blood vessel is cut open. Surgeon A cannot stop the bleeding using the robot. A support surgeon in the operating room (surgeon B) steps in and controls the bleeding. Despite this, the patient loses blood and is harmed.

Who is primarily responsible in this situation?

- Surgeon A
- Surgeon B
- Robot manufacturer
- Hospital
- Other

Case 2

Level 1: Human-controlled robot system

A surgeon uses a robotic telescope while operating on a patient. Its purpose is to inform the surgeon about the location of an important blood vessel. The surgeon plans to use this information and their knowledge of anatomy to perform the operation safely. During surgery, the robot malfunctions. It gives the surgeon inaccurate information. The blood vessel is cut and the patient is harmed.

Who is primarily responsible in this situation?

- Surgeon
- Robot manufacturer
- Hospital
- Other

Case 3

Level 2: Robot-assisted system

A patient has an operation where screws are inserted into the bone of their spine by a robot. A surgeon pre-programmes the robot with directions for the screws to be fixed. The robot then carries out the operation independently as the surgeon supervises. After the operation, the patient wakes up and cannot move their legs. A follow-up scan shows that a screw has been put into the wrong place, causing spinal injury. An investigation finds the surgeon had correctly programmed the robot, directing the screws away from the spinal cord.

Who is primarily responsible in this situation?

- Surgeon
- Robot manufacturer
- Hospital
- Other

Case 4

Level 3: Autonomous robot system
A surgeon recommends a hip replacement operation for a patient. A robot carries out the surgery independently and the surgeon, who supervises, does not intervene. The operation is technically successful and follow-up scans show that the hip was repaired as planned. However, the patient is left with worse hip pain, which badly affects their quality of life.

Who is primarily responsible in this situation?

- Surgeon
- Robot manufacturer
- Hospital
- Other

Case 5

Level 3: Autonomous robot system
An intelligent robot develops a new surgical technique to treat pancreatic cancer. Research through clinical trials shows the new technique is better than existing treatments. A surgeon refers a patient with newly diagnosed pancreatic cancer for the procedure. During the operation, the robot cannot manage a complication in the surgery and the patient is harmed.

Who is primarily responsible in this situation?

- Surgeon
- Robot manufacturer
- Hospital
- Other

How many cases were presented in this survey?

- 1
- 5
- 10
- 25

Thank you very much for taking part
